# A highly stable laccase obtained by swapping the second cupredoxin domain

**DOI:** 10.1038/s41598-018-34008-3

**Published:** 2018-10-23

**Authors:** Isabel Pardo, David Rodríguez-Escribano, Pablo Aza, Felipe de Salas, Angel T. Martínez, Susana Camarero

**Affiliations:** 1 Centro de Investigaciones Biológicas, CSIC, Madrid, Spain; 2 Present Address: National Bioenergy Center, National Renewable Energy Laboratory, Golden, CO USA

## Abstract

The robustness of a high-redox potential laccase has been enhanced by swapping its second cupredoxin domain with that from another fungal laccase, which introduced a pool of neutral mutations in the protein sequence without affecting enzyme functionality. The new laccase showed outstanding stability to temperature, pH (2–9) and to organic solvents, while maintaining the ability to oxidize high-redox potential substrates. By engineering the signal peptide, enzyme secretion levels in *Saccharomyces cerevisiae* were increased, which allowed to purify the engineered enzyme for further characterization. The purified domain-swap laccase presented higher activity in the presence of ethanol or methanol, superior half-lives at 50–70 °C, improved stability at acidic pH, and similar catalytic efficiency for DMP albeit a lower one for ABTS (due to a shift in optimum pH). A new N-glycosylation site and a putative new surface salt-bridge were evaluated as possible determinants for the improved stability by site-directed mutagenesis. Although neither seemed to be strictly responsible for the improved thermostability, the new salt bridge was found to notably contribute to the high stability of the swapped enzyme in a broad pH range. Finally, the application potential of the new laccase was demonstrated with the enzymatic treatment of kraft lignin, an industrially relevant lignin stream, at high temperature, neutral pH and short incubation times.

## Introduction

Laccases, belonging to the superfamily of blue-multicopper oxidases, are enzymes capable of oxidizing a broad range of phenols, aromatic amines and other chemical compounds with the concomitant reduction of molecular oxygen to water. They are typically monomeric proteins that consist of three cupredoxin domains (D1, D2 and D3) and present four catalytic copper ions: one T1 copper which accepts one electron from the reducing substrate; and one T2 and two T3 coppers that form a trinuclear cluster and transfer four electrons to O_2_ to render two molecules of H_2_O. Highly conserved residues (ten His and one Cys) located at D1 and D3 coordinate the copper atoms and provide a fast electron transfer pathway from T1 Cu to the trinuclear cluster^1^. While the redox potential at T1 Cu site defines whether a certain compound may be oxidized by laccase, effective oxidation is also determined by steric limitations and favorable interactions between the substrate and residues delimiting the enzyme’s substrate binding pocket, located in D1 and D2^2–4^.

For many years the engineering of laccases for their application in different fields has raised a great interest. Highly efficient enzymes that are active and stable under harsh operational conditions are needed to be used as industrial biocatalysts^5,6^. Directed evolution is a powerful tool for tailoring enzymes *a la carte*, allowing the attainment of desired properties in a few rounds of mutagenesis, screening and selection. However, after several generations of “uphill walk” on the protein fitness landscape, one may fail to obtain new single beneficial mutations, frequently caused by a trade-off between protein activity and stability^7,8^. Introduction of neutral mutations on the other hand may increase the protein’s robustness, while maintaining its function, and allow the accommodation of new beneficial mutations by an epistatic effect^9^. Indeed, the introduction of neutral mutations in directed evolution experiments, resembling natural genetic drift, has been successfully used to obtain more active proteins with increased stability or to enhance promiscuous enzymatic activities^10–12^. However, the simplest way to obtain functional sequences with an elevated number of neutral mutations (which may be hard to find and select in libraries generated by error-prone PCR) is the recombination of related sequences, rendering a rapid exploration of a large sequence space^13^. The recombination of DNA sequences can be performed randomly, either by sequence-dependent (DNA shuffling, StEP)^14,15^ or independent approaches to avoid biases in cross-over points (ITCHY, SCRATCHY, SHIPREC)^16–18^; and rationally, selecting cross-over points based on the inspection of protein structure and trying to minimize the disruption of interactions between residues (SCHEMA)^19–21^.

Previously we have described the engineering of chimeric laccase variants by random DNA shuffling of two high-redox potential laccases actively expressed in *Saccharomyces cerevisiae*^22^. Parent-types used came from the directed evolution of laccases from basidiomycetes *Pycnoporus cinnabarinus* (PcL, evolved variant 3PO) and *Coriolopsis* sp. PM1 (PM1L, evolved variant OB1)^23,24^. The selected chimeras presented an array of different properties compared to the parent-types, including shifted optimum pH and increased thermostability. The most stable chimeras were more similar to parent OB1, with relatively small sequence blocks of 3PO exchanged in D1 and D3. No crossover events were produced in D2, most probably due to the lower sequence identity of both laccases in this domain. In the present work, we describe the construction of a new hybrid laccase by structure-guided DNA shuffling to replace the D2 of OB1 laccase for that of 3PO laccase. The domain-swap laccase presents a remarkable increase in stability to high temperature, acidic pH and organic co-solvents. Possible causes for this enhancement are evaluated, and the application potential of this new laccase is demonstrated with the enzymatic treatment of kraft lignin.

## Results and Discussion

### Construction and preliminary characterization of the domain-swap laccase

**Figure 1:**
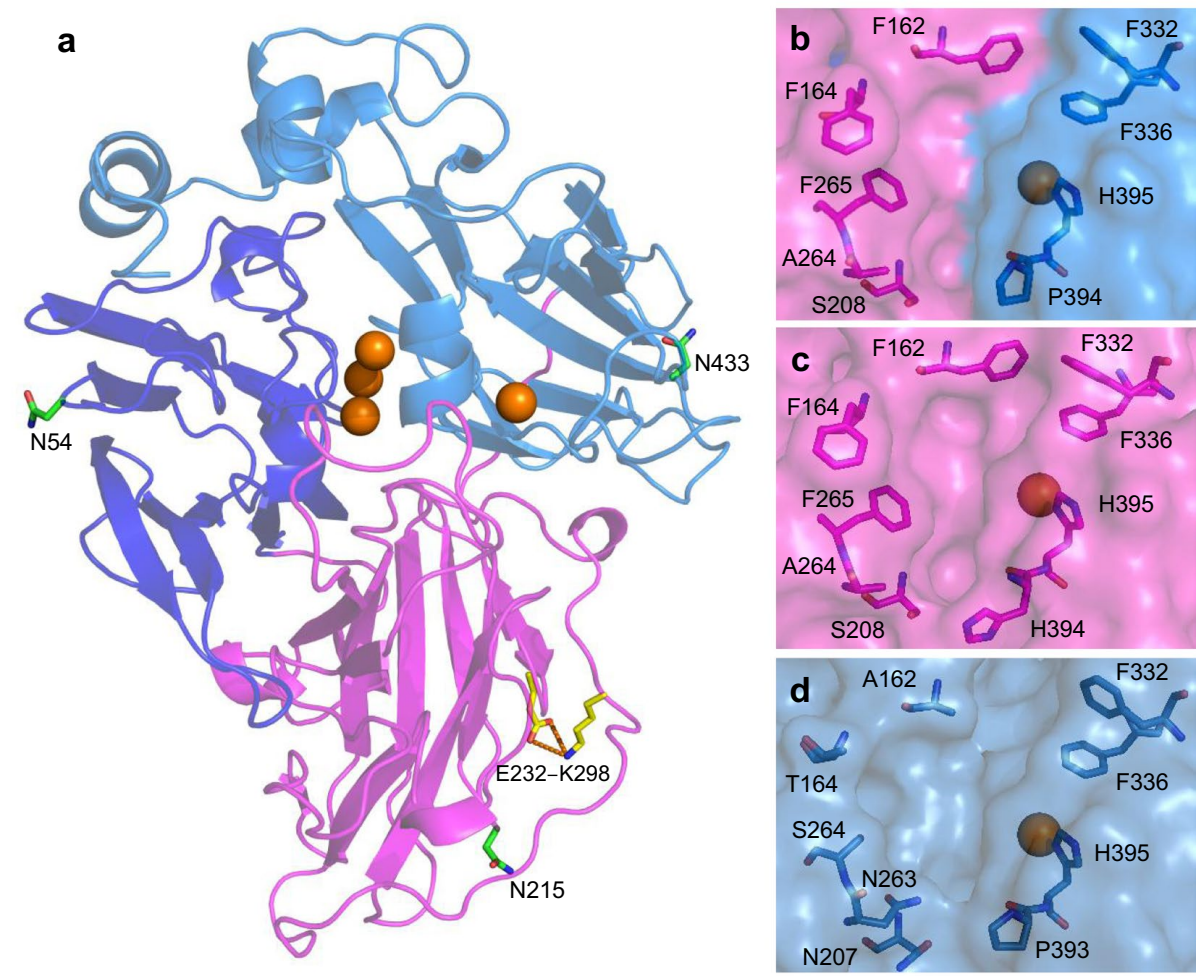
Structure model of domain-swap laccase. (**a**) Cartoon representation showing N-glycosylation sites as green sticks and putative salt bridge inherited from 3PO-D2 as yellow sticks. D1 and D3, inherited from OB1, are shown in dark and light blue, respectively. D2, inherited from 3PO, is shown in magenta. Catalytic coppers are shown as spheres. (**b**–**d**) Close-ups of the substrate binding pockets of domain-swap laccase (**b**), 3PO (**c**) and OB1 (**d**). Residues delimiting the substrate binding pocket are shown as sticks.

We decided to exchange D2 from OB1 for that of 3PO, as previous random chimeragenesis had shown that chimeric laccases more similar to OB1 were more active and stable^22^. The sequence identity between both laccases for this domain is only 69%, as compared to 81% for D1 and 79% for D3. Based on the crystal structures of PM1L and PcL, which are the wild-type fungal laccases from which OB1 and 3PO were respectively evolved, cross-over points were selected in loops between the three cupredoxin domains in order to maintain their integrity, specifically between residues 129–130 and 312–313 (313–314 according to 3PO numbering) (Fig. 1a). Since the optimum temperatures for maximal production of both parent-type laccases are different, the production of the domain-swap laccase was evaluated at 20 and 30 °C. A maximum activity of 118 U/L was reached at 20 °C for this variant (see Supplementary Fig. S1). However, this value was much lower than the levels previously obtained for either parent-type: 300 U/L for 3PO at 20 °C and 1000 U/L for OB1 at 30 °C^23,24^. Nevertheless, the structure-guided recombination strategy followed here allowed us to obtain an active enzyme despite the high number of mutations introduced (65 substitutions in a 185 amino acid sequence-length).

**Figure 2:**
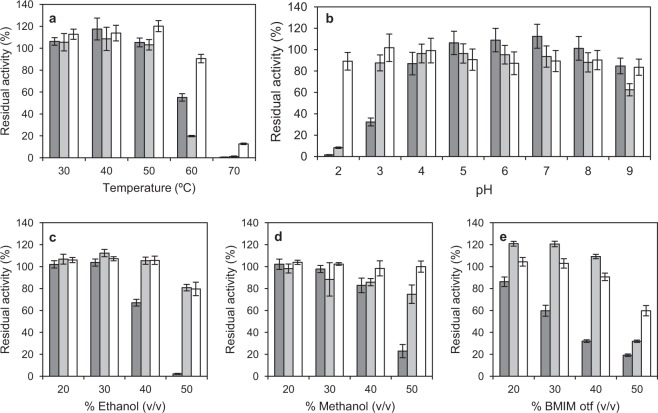
Long-term stabilities of domain-swap laccase and OB1 and 3PO parent-type laccases. Residual activities after 6-h incubation at different temperatures (**a**), pH (**b**) or solvent concentrations including ethanol (**c**), methanol (**d**) or BMIM otf (**e**). From left to right: OB1, 3PO and domain-swap laccases. Error bars indicate standard deviation for triplicates.

For the preliminary characterization of the domain-swap laccase we used concentrated supernatants. The swapped laccase showed a T_50_ (10 min) value 7 °C higher than that of OB1 and 3PO parent-types (Table 1, Supplementary Fig. S2). It also showed a remarkable increment in long-term stability (6 hours) at 50–70 °C with respect to OB1 and 3PO laccases (Fig. 2a). Furthermore, the new enzyme resulted quite more stable at pH 2 (Fig. 2b). This was in accordance with a shifted pH optimum towards more acidic values for the oxidation of 2,2′-azinobis-(3-ethylbenzothiazoline-6-sulfonic acid) (ABTS), although optimum pH for the oxidation of 2,6-dimethoxyphenol (DMP) was unaltered (Supplementary Fig. S3a,b). What is more, when the incubation period was prolonged up to 24 h, the greater stability of the swapped laccase in the whole pH range with respect to the parent laccases was accentuated. It still retained 80–90% the initial activity after 24 h at pH 3–9 and >30% at pH 2 (see Supplementary Fig. S4). Regarding stability to solvents (Fig. 2c–e), we found that both parent-types and the domain-swap variant were stable in up to 50% (v/v) acetone and DMSO (data not shown). However, OB1 parent-type was severely inactivated after 6 h in 50% ethanol and methanol and was more sensitive to the ionic liquid 1-butyl-3-methylimidazolium trifluoromethanesulfonate (BMIM otf). In contrast, the domain-swap variant was quite stable in 20–50% ethanol, methanol and BMIM otf. Surprisingly, the activity of 3PO was enhanced after the 6-h incubation in up to 40% BMIM otf. Although in general liquid ions have a negative effect on laccase activity, an increased activity and stability of *Trametes versicolor* laccase in the presence of 13 different ionic liquids has also been described in the literature^25,26^. Finally, the long-term stability of the domain-swap laccase was compared with those of three laccase variants (namely 3A4, 7A12 and 7D5) that had been selected because of their high thermostability from the random chimeragenesis library previously obtained from the same parent-types^22^. The domain-swap laccase resulted remarkably more stable than these random chimeric variants to high temperature and acid pH, whereas the stability to organic solvents was similar in all cases (see Supplementary Fig. S5).

**Table 1:** T_50_ (10 min) values for parent-type laccases (OB1 and 3PO), domain-swap laccase (swap) and variants of domain-swap laccase obtained by site-directed mutagenesis.

Variant	T_50_ (10 min) (°C)
OB1	71.4 ± 0.3
3PO	70.8 ± 0.2
Swap	78.1 ± 0.6
Swap-N215G	78.6 ± 0.2
Swap-E232T/K298Q	77.3 ± 0.2

### Enhanced secretion and biochemical characterization of the domain-swap laccase

Next, we sought to enhance the production of the domain-swap laccase in yeast. Secretion of the domain-swap laccase is mediated by an evolved alpha-factor signal peptide (α_OB1_) that was obtained during the directed evolution of PM1L^24^. To evaluate whether production levels could be increased, we tested two other mutant alpha-factor signal peptides that had been obtained in different directed evolution campaigns of basidiomycete laccases in yeast: α_3PO_^23^ and α_PK2_ (with four mutations of difference with respect to α_3PO_, unpublished data). Signal peptide α_PK2_ was found to increase laccase activity almost 2-fold in the culture media (over 250 U/L). This result and previous works demonstrate that some mutations in the alpha-factor pre-proleader sequence can be exchanged between laccases to enhance their heterologous secretion in yeast, which is of interest for the large-scale production of these enzymes and to tackle the limitations of low secretion levels in future protein engineering efforts^24,27^.

Partial purification of the domain-swap laccase showed that it was produced in two isoforms with different glycosylation levels: one hyperglycosylated and the other with 10–15% glycosylation (Supplementary Fig. S6a). However, only the latter (Mw ~50 kDa) could be purified to homogeneity for biochemical characterization. The new variant presented lower *k*_cat_ for DMP or ABTS at pH 5 than those of parent-types. However, a 2-fold lower *K*_M_ for DMP resulted in a similar catalytic efficiency for this substrate, whereas a *K*_M_ equal to that of 3PO for ABTS resulted in a 3–5 fold diminished catalytic efficiency (Table 2). This could be an effect of the shifted pH-activity profile towards more acid values for ABTS (40% activity of the domain-swap laccase at pH 5 against >80% activity of the parent laccases, Supplementary Fig. S3). To confirm that the lower *k*_cat_ values were not an effect of a decreased redox potential, we compared the oxidation of vanillin, Evans Blue dye and violuric acid (with oxidation potentials between 0.85 and 1.0 V vs SHE)^28–30^ by the domain-swap laccase and both parent-types (see Supplementary Fig. S7). The fact that the domain-swap laccase oxidized these high redox potential substrates (even better than 3PO parent) evidenced it maintained the high-redox potential of the parent-types, although the three laccases presented dissimilar oxidation rates for the different substrates.

**Table 2:** Kinetic constants for purified domain-swap laccase and parent-types OB1 and 3PO.

Variant	Substrate	*K*_m_ (mM)	*k*_cat_ (s^−1^)	*k*_cat_/*K*_m_ (mM^−1^ s^−1^)
Swap	ABTS	0.027 ± 0.003	166 ± 6	6160 ± 726
	DMP	0.084 ± 0.003	82 ± 1	986 ± 33
OB1^1^	ABTS	0.006 ± 0.001	200 ± 7	31746 ± 5406
	DMP	0.140 ± 0.020	134 ± 5	957 ± 141
3PO^2^	ABTS	0.024 ± 0.002	483 ± 10	19944 ± 1713
	DMP	0.213 ± 0.013	197 ± 3	923 ± 58
Swap-P394H^*^	ABTS	0.006 ± 0.001	n.d	—
	DMP	0.89 ± 0.037	n.d	—

^1^From Maté *et al*., 2010^24^. ^2^From Camarero *et al*. 2012^23^. ^*^Crude enzyme.

It is worth mentioning that the substrate-binding pocket of the domain-swap laccase resembles that of 3PO parent-type because some of the less conserved residues delimiting the entrance of the substrate binding pocket in basidiomycete laccases – namely residues 162, 164, 264 and 265 – were exchanged in the new variant (Fig. 1b–d). Given that the chemical nature of the amino acids delimiting the substrate binding pocket determines laccase activity^4,31^, akin kinetic constants could be expected for the domain-swap laccase and 3PO parent-type. However, a single-point mutation in the active site may exert a significant effect on laccase activity^2,3,32^, and in fact the T1 site environments of the domain-swap and 3PO laccases are not the same. Actually, the domain-swap laccase inherits mutation N208S but not mutation P394H (adjacent to H395 coordinating the T1 copper) from 3PO parent. Both mutations were shown to be crucial for the enhanced activity of 3PO by computational simulation^31^. Together they provide a new substrate binding mode in 3PO laccase that favors the electron transfer to T1 copper, thus raising the oxidation rates respecting wild-type PcL^31^. Nevertheless, while this new and better oxidation site is preferentially occupied by large substrates such as ABTS, small substrates like DMP can simultaneously (and competitively) occupy both the new site and the original site found in the wild-type PcL, thus resulting in a lower affinity for DMP in 3PO laccase^23,31^. Albeit the domain-swap laccase shows a lower *k*_cat_ than 3PO, its catalytic efficiency oxidizing DMP is similar, precisely due to the lower *K*_M_. We designed the swap-P394H variant to assess the contribution of mutations N208S and P394H to substrate affinity. This resulted in a 10-fold increment of *K*_M_ for DMP and a lower *K*_M_ for ABTS (Table 2). Thus, both mutations seem to have the same effect as in 3PO laccase, creating a new substrate binding mode which could be occupied by DMP at the same time than the original mode (raising *K*_M_), whereas it would be preferred by ABTS (improved affinity). In addition, the lack of P394H mutation in the domain-swap laccase would explain the more acidic optimum pH of this variant towards ABTS, as demonstrated by the shifted optimum pH for this substrate in the swap-P394H variant. The same effect was observed when introducing P394H mutation in PcL wild-type, changing the activity profile of PcL to a less acidic one (similar to 3PO’s) (see Supplementary Fig. S3c,d).

**Figure 3:**
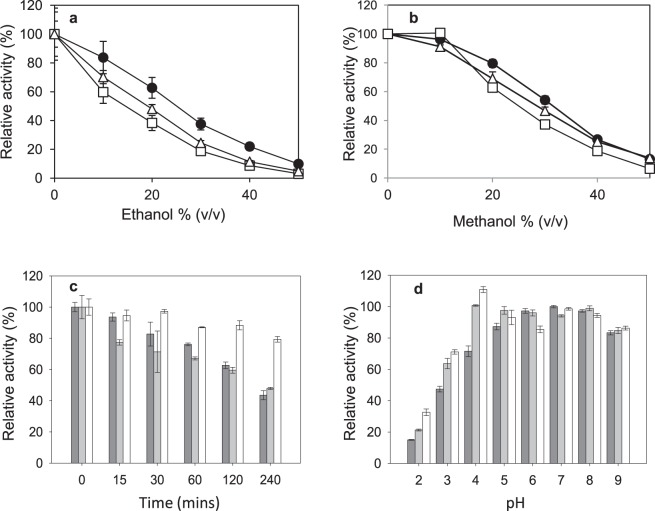
Activity at different concentrations of ethanol (**a**) or methanol (**b**) of purified domain-swap laccase (black circles) compared with purified wild-type parent-types PcL (white triangles) and PM1L (white squares); and stabilities in 40% methanol at different times (**c**) and after 6 h incubation at different pH (**d**) of purified domain-swap and OB1 and 3PO parent laccases. Bars from left to right: purified OB1, 3PO and domain-swap laccases.

Finally, we determined the effect of temperature, pH and organic solvents in the activity and stability of the purified domain-swap laccase. It presented maximum activity around 65–70 °C, similarly to the parent-types (Supplementary Fig. S8). However, thermal inactivation kinetics (Supplementary Fig. S9) showed significantly lower inactivation constants and superior half-lives for the swap laccase at 50–70 °C when compared to the parent-types (Table 3). We calculated the activation energy (Ea) from the corresponding Arrhenius plots as a measure of how the rate of inactivation varies with temperature. The lower E_a_ for the inactivation of the domain-swap laccase (127 kJ/mol) respecting those of the parent-types (165 kJ/mol for OB1 and 186 kJ/mol for 3PO) reflected a lesser sensitivity of the new laccase to temperature change^33^. All these data confirm the notable enhancement in thermostability by domain-swapping. In addition, the purified enzyme showed high tolerance to the presence of organic solvents, presenting around 40% activity in 30% ethanol (v/v) and around 55% activity in 30% methanol (v/v). These values are significantly better than the activities of the purified wild-type laccases from PM1 and *P. cinnabarinus* (Fig. 3a,b). Moreover, the domain-swap laccase retained over 80% activity after 3 h in 40% methanol as opposed to 40% residual activity of parent laccases under the same conditions (Fig. 3c). Finally, the new variant showed enhanced stability in acidic pH (2–4) compared to the parent laccases, while maintained the stability at basic pH (Fig. 3d). All these results confirm the superior robustness of the variant designed in this study.

**Table 3:** Thermal inactivation constants and half-life values for purified domain-swap laccase and parent-types OB1 and 3PO.

Temperature (°C)	Swap		OB1		3PO		Swap-hgly	
	*k* (h^−1^)	t_1/2_ (h)	*k* (h^−1^)	t_1/2_ (h)	*k* (h^−1^)	t_1/2_ (h)	*k* (h^−1^)	t_1/2_ (h)
50	0.05	13.8	0.086	8.1	0.195	3.6	0.055	12.6
60	0.195	3.5	0.44	1.6	1.098	0.6	0.215	3.2
70	0.798	0.9	3.7	0.2	7.072	0.1	0.668	1.0

Values for hyperglycosylated isoform of domain-swap laccase (swap-hgly) are also provided for comparison.

### Evaluation of the factors determining stability in the domain-swap laccase

It is noteworthy that even though the mechanisms of protein inactivation by high temperature, pH or co-solvents are different, high stability to all of them converge in the domain-swap laccase, in contrast to what is found in the parent-types (i.e. OB1 is more thermostable but less resistant to high concentrations of organic solvents or acid pH than 3PO). The burying of hydrophobic residues in the folded protein core, the formation of disulfide bonds and the occurrence of polar interactions in the protein surface – such as salt bridges and hydrogen bonding between amino acids or between amino acids and sugar moieties in glycoproteins – determine enzyme stability to these environmental factors. However, the extent to which each of these individual interactions contributes to total stability is difficult to assess and largely depends on the protein studied^34^. In order to identify some new interactions that could contribute to the increased stability of the domain-swap laccase, we searched for differences in N-glycosylation and superficial salt-bridges between the structural models of the domain-swap variant and both parent-types. No differences in cysteine bonding were expected, as both parent-types present two disulfide bonds (connecting D1-D2 and D1-D3) whose positions are extremely conserved in all basidiomycete laccases.

Concerning glycosylation, the domain-swap laccase presents a new N-glycosylation site (N215) inherited from 3PO-D2 in addition to the two sites (N54 and N433) found in OB1 parent (Fig. 1a). N-glycosylation sites N54 and N433 are highly conserved amongst fungal laccases, and are considered to play an important role during nascent protein folding and protein stabilization^34–36^. To assess the role of the additional glycosylation site N215 in the domain-swap laccase, we removed it by mutating this residue for the glycine found in OB1. When we performed SDS-PAGE of non-purified domain-swap and swap-N215G laccase variants stained with DMP (non-denaturalized samples), we found equal electrophoretic mobility for the swap-N215G variant and the non-hyperglycosylated isoform of domain-swap laccase (see Supplementary Fig. S6b). Thus, N215 site seems to be responsible for the hyperglycosylation of the domain-swap laccase. The involvement of N215 site in hyperglycosylation of laccase is corroborated by the fact that 3PO laccase is 50% hyperglycosylated^23^, whereas OB1 laccase (lacking N215 site) is not (10% glycosylation)^24^. Then, we evaluated the possible contribution of hyperglycosylation in N215 site to enzyme’s stability to temperature and pH by comparing the domain-swap and swap-N215G laccases. Neither the T_50_ (Table 1) nor the residual activity of the enzyme after 6 h incubation at different pH (see Supplementary Fig. S10) were affected by the removal of N215 site. Moreover, we double-checked the effect of the hyperglycosylation in the superior behavior of the domain-swap laccase at high temperature by comparing the two isoforms (hyperglycosylated and non-hyperglycosylated). The close similarity of their optimal temperatures (see Supplementary Fig. S8) as well as thermal inactivation constants and half- lives at 50–70 °C (Table 3) confirmed that the enhanced thermostability of the domain-swap laccase is not caused by extra glycosylation of the enzyme.

Next, putative salt-bridges on the protein surface were inspected, searching for acid-basic amino acid pairs with <5 Å distance between them in the protein models for the domain-swap laccase and OB1 and 3PO parent laccases. The three enzymes presented 13 putative surface salt bridges in total. However, the domain-swap variant inherited acid-basic pair E232–K298 from 3PO-D2 (not present in OB1-D2), which could form a new salt bridge (Fig. 1a, Supplementary Table S3). To assess the contribution of this new salt bridge to the increased stability of the domain-swap laccase, we reverted these two residues to those originally present in OB1 laccase, obtaining the swap-E232T/K298Q variant. Laccase activity obtained during production was severely decreased (only 5 U/L in the shake-flask). The swap-E232T/K298Q variant presented a T_50_ value only 1 °C lower than that of the domain-swap laccase (Table 1). However, the contribution of the salt bridge was more evident with regards to stability of the enzyme to pH. The swap-E232T/K298Q variant showed a notable decrease in stability at pH 8–9 and 2–3 with respect to the domain-swap laccase (Supplementary Fig. S4).

Taking these results into account, other new interactions introduced by replacing D2, which would be difficult to pin-point by site-directed mutagenesis approaches, would most probably also contribute to the outstanding stability of the new laccase. More complex calculations, such as those obtained by molecular dynamics simulations, could help find possible hot-spots for further evaluation.

### Enzymatic treatment of kraft lignin

**Figure 4:**
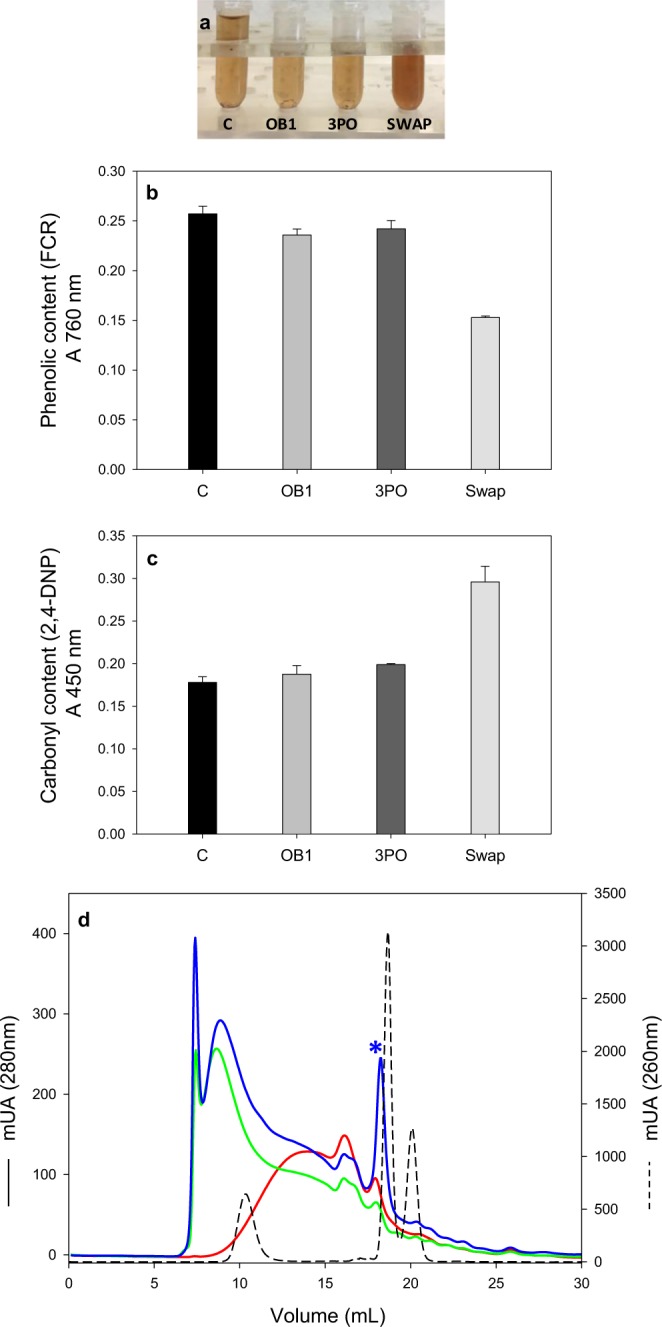
Laccase treatment of kraft lignin. (**a**) From left to right, kraft lignin treated for 1 h at 65 °C, pH 6, without laccase (C) or with OB1, 3PO or domain-swap laccases. (**b**) Phenolic content of treated kraft lignin as determined by A760 nm of the FCR assay. (**c**) Carbonyl content of treated kraft lignin as determined by A 450 nm of the 2,4-DNP assay. (**d**) SEC profile of kraft lignin before (red line) and after treatment with OB1 (green line) or domain-swap (blue line) laccases. Dashed line (from left to right): polystyrene sulfonate (4210 Da), syringaresinol (418 Da) and sinapic acid (224 Da) used as standards (Abs. 260 nm).

The thermoactivity and thermostability of the domain-swap laccase make it a suitable catalyst for application, as shown by its superior performance during the enzymatic treatment of industrial kraft lignin when compared to parent laccases. Kraft lignin obtained from the black liquors of the kraft pulping process is characterized by its high recalcitrance and insolubility in water which is a major handicap for the enzymatic treatment^37^. However, lignin was notably solubilized by laccase after 1 h of treatment at pH 6 and 65 °C compared with a control without enzyme at the same conditions. Kraft lignin solubilization by the domain-swap laccase was suggested by less precipitated lignin observed and the darker color of the supernatant (Fig. 4a), and lignin transformation was demonstrated by the changes in the phenolic and carbonyl contents and in the molecular weight distribution of solubilized lignins (Fig. 4b–d). Only the domain-swap laccase produced an important modification in lignin phenolic content, measured by the increase in A_760_ after reaction with Folin-Ciocalteau reagent (FCR)^38,39^. This decrement in phenolic content coincided with a significant increment in carbonyl content caused by the oxidation of lignin-units side-chains by laccase, as determined by the increase in A_450_ of the phenylhydrazone derivates produced by the reaction of the benzylic aldehyde or ketone groups with 2,4-dinitrophenylhydrazine (2,4-DNP)^40^.

Finally, an increment of the average molecular weight (revealing lignin polymerization) and a higher amount of solubilized material analyzed by SEC, were observed after laccase treatment. Again, changes in lignin’s molecular weight distribution profile were more marked after treatment with domain-swap laccase. It is worth noting that an important peak corresponding to lower molecular weight species (due to lignin depolymerization), appeared exclusively after treatment with the domain-swap laccase (asterisk in Fig. 4d). All these results indicate that competing depolymerization and repolymerization/grafting reactions may be taking place^41^. The increased water solubility suggests enzymatic fractioning of the lignin polymer^37^ and the strong increment in carbonyl content suggests superior oxidation of lignin-unit side-chains. Conversely, the significant reduction of lignin phenolic content and the general increase of molecular mass would be caused by subsequent coupling reactions between the oxidized lignin monomers/oligomers.

In summary, kraft lignin was strongly solubilized and activated by oxidation with the new laccase at neutral pH and 65 °C, thanks to its optimal activity at the reaction temperature and to its high thermostability, making feasible the release of small lignin oligomers at short reaction times. This points out the potential of the new laccase as biocatalyst for kraft lignin valorization.

## Conclusions

The applicability of a fungal laccase is raised here by increasing its robustness to extreme reaction conditions. Outstanding stability to temperature, to acid and basic pH, and to the presence of organic solvents amazingly converge in the new laccase engineered by exchanging the second cupredoxin domain with that from another high-redox potential laccase. One new surface salt-bridge significantly contributes to raise the enzyme’s stability towards acid pH. However, other new interactions within the pool of neutral mutations brought in by domain-swapping should be responsible for the overall robustness of the enzyme. Laccase activity was not significantly compromised by the remarkable enhancement in stability, making the new enzyme a promising scaffold for future directed evolution campaigns, as now it could be more tolerant to the acquisition of new mutations.

## Material and Methods

### Generation of 3D-structure protein models

A protein model of the swapped laccase was generated with Modeller in the UCSF Chimera graphical interface^42,43^. Crystal structures of laccases from basidiomycete PM1 (PDB 5ANH) and *P. cinnabarinus* (PDB 2XYB) were used as templates. Generated models with lowest Z-score were selected and visually inspected with PyMol Molecular Graphics System (Schrödinger, LLC).

### Construction of domain-swap mutant and site-directed mutagenesis

General considerations regarding PCR conditions, purification of amplification products and *in vivo* cloning in competent *S. cerevisiae* BJ5465 cells have been described previously^22^. The pJRoC30 plasmids carrying the 3PO (evolved PcL) and OB1 (evolved PM1L) constructions, fused to their respective evolved alpha-factor prepro-leaders, were obtained in previous works^23,24^. Chimeric primers with overhangs of 10–20 bp complementary to the adjacent cupredoxin domain from the other parent were used for the amplification of each domain (Supplementary Table S1). The PCR products were mixed in equimolar proportion and reassembled by PCR overlap extension and subsequently amplified as described previously^22^. Cloning of the whole reassembled coding DNA sequence (CDS) into the pJRoC30 vector (previously linearized with NotI and BamHI) was done *in vivo* by designing overhangs which specifically anneal with the ends of the linearized vector (annealing regions of 40 bp and 66 bp). CDS products (400 ng) were co-transformed with linearized pJRoC30 vector (100 ng) into competent BJ5465 cells with the Yeast Transformation Kit (Sigma). Transformed cells were then grown on Synthetic Complete (SC) drop-out plates (6.7 g/L YNB, 1.92 g/L drop-out media supplements without uracil, 20 g/L glucose, 20 g/L agar, 25 mg/L chloramphenicol) at 30 °C for 2–3 days, until colonies containing the whole autonomously replicating vector appeared. Plasmid was then extracted, amplified in *E. coli* DH5α cells and purified for sequencing in order to confirm correct construction. Site-directed mutants were obtained by *In Vivo* Overlap Extension^44^, designing complementary mutagenic primers with ∼20 bp flanking the desired mutations (Supplementary Table S2).

### Laccase production and purification

Transformed yeast clones were cultured in expression medium (20 g/L peptone, 10 g/L yeast extract, 20 g/L galactose, 60 mM phosphate buffer pH 6.0, 4 mM CuSO_4_, 25 g/L ethanol, 25 mg/L chloramphenicol) for laccase production, as described previously^23^. Laccase activity in the culture supernatant was followed spectrophotometrically with 3 mM 2,2′-azino-bis(3-ethylbenzothiazoline-6-sulfonic acid) (ABTS, ε_418_ = 36,000 M^−1^ cm^−1^) in 100 mM acetate buffer pH 5, defining one activity unit (U) as the amount of enzyme needed to transform 1 µmol substrate/minute. When maximum activity was reached, cells were separated by centrifugation at 10,000 *g*, 4 °C, and supernatants were filtered through 0.8 and 0.45 µm pore-size membranes and then concentrated by ultra-filtration through 10,000 MWCO membranes. Finally, laccase purification by high-performance liquid chromatography (HPLC) was performed as described previously, using two anion-exchange and one molecular exclusion steps^3^. All chromatographic steps were carried out in 20 mM Tris-HCl buffer, pH 7, with NaCl.

### Characterization of domain-swap laccase

In general, enzymatic assays for the preliminary characterization of the new hybrid laccase were carried out in 96-well plates using ~0.1 U/mL dilutions of unpurified, concentrated culture supernatant and measuring laccase activity in a Spectramax Plus384 plate reader (Molecular Devices, UK). T_50_ (10 min) value and pH activity profiles towards ABTS and 2,6-dimethoxyphenol (DMP, ε_469_ = 27,500 M^−1^ cm^−1^) were determined as described previously^22^. For the long-term stability assays, dilutions were prepared in 20 mM Tris-HCl buffer, pH 7 (temperature and solvent assays), or 100 mM Britton and Robinson (B&R) buffer, pH 2–9, for the pH assay. In the solvent stability assay, ethanol, methanol, acetone, dimethyl sulfoxide (DMSO) or 1-butyl-3-methylimidazolium trifluoromethanesulfonate (BMIM otf) were added to a final concentration of 20, 30, 40 or 50% (v/v). In the case of BMIM otf, plates were under constant agitation during incubation time to assure homogenous mixture of the sample. Sample plates were incubated at room temperature for pH and solvent assays or in the thermocycler at 30, 40, 50, 60 and 70 °C for the temperature assay. In all cases, after a 6-h incubation, residual activities were measured in triplicate by transferring 20 μL aliquots to new 96-well plates and adding 180 μL 3 mM ABTS in 100 mM sodium acetate buffer, pH 5.

Kinetic constants towards ABTS and DMP for the purified laccase and concentrated supernatants were also obtained in the plate reader. Concentration ranges used were 0.00125 mM to 1 mM for ABTS, and 0.0025 to 9.6 mM for DMP. All measurements were carried out at 25 °C in 100 mM acetate buffer, pH 5, following the increase in absorbance at the corresponding wavelength during 5 min. Initial rates for each substrate concentration were obtained from the linear portion of the curve, as described previously^23^.

Optimum temperature for the purified laccase was determined in the spectrophotometer with a Peltier temperature control using 3 mM ABTS in 100 mM acetate buffer pH 5 (triplicate samples). The oxidation was followed during the first min of reaction with the substrate pre-incubated at the corresponding temperature. Thermal inactivation kinetics were carried out by incubating 0.1 U/mL enzyme at 50, 60 and 70 °C in 20 mM Tris HCl buffer pH 7. Triplicate 20 µL samples were taken at different periods of time and the activity was measured with the substrate aforementioned in the plate reader at room temperature after chilling the enzyme during 10 min on ice. Activity in the presence of ethanol and methanol was determined in the plate reader using 20 µL of ~0.1 U/mL dilutions of purified laccase and adding 3 mM ABTS in 100 mM acetate buffer, pH 5, and 0, 10, 20, 30, 40 or 50% (v/v) co-solvent to a final volume of 200 µL.

### Oxidation of kraft lignin

Eucalypt kraft lignin (ENCE, Spain) (1 g/L) was treated with 0.1 U/mL of unpurified laccase in 50 mM citrate phosphate buffer pH 6, in 30 mL final volume. After 1 h of reaction at 65 °C with gentle agitation, 2-mL samples were centrifuged for 10 min at 10,000 *g*. Lignin subjected to same procedure but without enzyme addition was used as control. The phenolic content in supernatants was measured with the Folin–Ciocalteau reagent (FCR) following the protocol described previously with minor modifications^39^. In it, 5 µL lignin samples were mixed for 5 min in 96-well plates with 30 µL 0.135–0.165 M FCR and water to a final volume of 200 μL. Then, 50 µL Na_2_CO_3_ (20% w/v) were added and plates were incubated with gentle agitation until the development of blue color (around 1 h) when absorbance at 760 nm was measured in the plate reader. Lignin carbonyl content was determined by the 2,4-dinitrophenylhydrazine (2,4-DNP) assay^36^. In this case, 20 µL lignin samples were mixed with 30 µL 100 mM HCl and 50 µL 1 mM 2,4-DNP dissolved in 100 mM HCl. Reactions were incubated at 25 °C for 10 min, after which 100 µL 1 N NaOH was added. Absorbance was measured at 450 nm in the plate reader. All measurements were carried out in triplicate. Changes in the molecular weight distribution of kraft lignin were measured by SEC chromatography. Two-hundred µl aliquots were loaded in a size exclusion Superdex Peptide 10/300GL column pre-equilibrated with 150 mM NaCl in 20 mM Britton and Robinson buffer pH 11.6 and coupled to an ÄKTA purifier system. An equimolar mix (1.6 mM) of sulfonated polystyrene (4,210 Da), syringaresinol (418 Da) and sinapic acid (224 Da) was used as standards. Lignin fractions and standards were eluted at 1 mL/min flow and monitored at 280 nm and 260 nm (polystyrene maximum absorbance).

## Electronic supplementary material

Supplementary Tables and Figures
