# Supplementary material for: A highly stable laccase obtained by swapping the second cupredoxin domain
Source: Sci Rep. 2018 Oct 23;8:15669. doi: 10.1038/s41598-018-34008-3 (PMC6199291; doi:10.1038/s41598-018-34008-3)
Supplement: Supplementary file 1 — Supplementary Tables and Figures [file 41598_2018_34008_MOESM1_ESM.pdf]

A highly stable laccase obtained by swapping the second cupredoxin domain

Isabel Pardo, David Rodríguez-Escribano, Pablo Aza, Felipe de Salas, Angel T. Martínez and Susana Camarero

SUPPLEMENTARY INFORMATION

Table S1. Primers used for construction of domain-swap laccase and for amplification of reassembled products. Sequences annealing with pJRoC30 are shown in italics, with 3PO underlined, and with OB1 in normal font. D1-OB1 was amplified with extpJR-F and D1-OB1 rev. D2-3PO was amplified with D2-3PO fwd and D2-3PO rev. D3-OB1 was amplified with D3-OB1 fwd and extpJR-R. The three amplification products were assembled and then further amplified by nested PCR with RMLN and RMLC. The latter two primers as well as extpJR-F and extpJR-R overlap with the ends of linearized plasmid.

**Primer**	**Forward/ Reverse**	**5' → 3' Sequence**
extpJR-F	F	*CTGGGGTAATTAATCAGCGAAGC*
extpJR-R	R	*CCAAAACCTTCTCAAGCAAGG*
D1-OB1 rev	R	<u>GTGAGGATCGTT</u>AGGGTCATAGACAAC
D2-3PO fwd	F	GTCTATGACCCT<u>AACGATCCTCACGC</u>
D2-3PO rev	R	GTCGGGTTGCC<u>AGGCACAGGCATAG</u>
D3-OB1 fwd	F	<u>CCTGTGCCT</u>GGCAACCCGACCC
RMLN	F	*CCTCTATACTTTAACGTCAAGG*
RMLC	R	*GGGAGGGCGTGAATGTAAGC*

Table S2. Primers used for site-directed mutagenesis of the domain-swap laccase. The mutated variants were obtained by using IVOE (In vivo overlap extension)[44]. Mutated codons are underlined. RMLN and RMLC primers (sequences overlapping with the ends of linearized plasmid) were used as sense primer for reactions with mutagenic antisense primers (RMLN) and as antisense primer for reactions with mutagenic sense primers (RMLC).

**Primer**	**5’ → 3’ Sequence**
N215G-F	GCCATACATTCAGCATTGATGGTCACACAATGACTATAATTGAGGC
N215G-R	GCCTCAATTATAGTCATTGTGTGACCATCAATGCTGAATGTATGGC
E232T-F	GGACTCGATCAACACTCAACCCCTAACTGTTGATTCAATCCAGATTTTTGC
E232T-R	GCAAAAATCTGGATTGAATCAACAGTTAGGGGTTGAGTGTTGATCGAGTCC
K298Q-F	CGTCTGTCCAGACTACTCCTACGCAGCCTCTGAACGAGGTCGACTTGC
K298Q-R	GCAAGTCGACCTCGTTCAGAGGCTGCGTAGGAGTAGTCTGGACAGACG
P394H-F	CCCGGCTTCCACCACCCCTTCCACTTG
P394H-R	CAAGTGGAAGGGGTGGTGGAAGCCGGG

Table S3. Putative surface salt bridges in parent-types and domain-swap laccase as predicted by PyMol. "<5 Å" indicates amino acid pairs for which PyMol does not predict hydrogen bonding but whose side-chains are less than 5 Å apart. The acid-basic pair inherited by the domain-swap laccase from 3PO domain is shown in bold. The corresponding non-charged residues (unable to form a salt bridge) in OB1 are shown in italic.

**OB1**			**3PO**			**SWAP**		
Acid	Basic	Bonded	Acid	Basic	Bonded	Acid	Basic	Bonded
D96	R43	YES	D96	R43	YES	D96	R43	YES
D118	R22	YES	D118	R22	YES	D118	R22	YES
D128	K40	<5 Å	D128	K40	<5 Å	D128	K40	<5 Å
D131	R196	YES	D131	R197	YES	D131	R197	YES
D138	R194	YES	D138	R195	NO	D138	R195	NO
D140	R198	YES	D140	R199	YES	D140	R199	YES
D213	R259	YES	D214	R260	YES	D214	R260	YES
D223	R422	YES	D224	R423	YES	D224	R423	YES
T231	*Q297*	NO	**E232**	**K298**	<5 Å	**E232**	**K298**	<5 Å
E287	R175	YES	E288	R176	YES	E288	R176	YES
D378	R439	YES	S379	E440	NO	D379	R440	YES
E380	R437	YES	E381	R438	YES	E381	R438	YES
D423	R242	YES	D424	R243	YES	D424	R243	YES
E457	K157	NO	D458	K157	<5 Å	D458	K157	<5 Å
D495	K59	YES	D496	K59	YES	D496	K59	YES

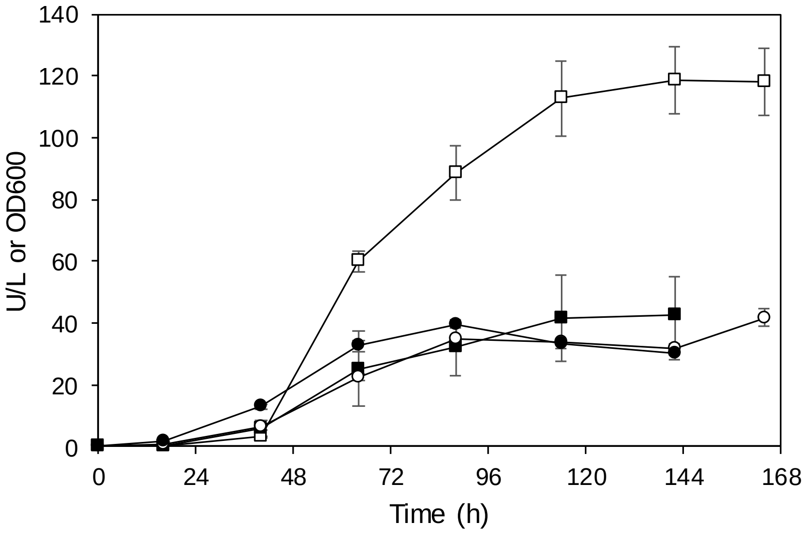

Figure S1. Time course for OD600 (circles) and laccase activity (squares) during the shake-flask production of domain-swap laccase at 20 (white) and 30 (black) ºC. Error bars indicate standard deviation for triplicates.

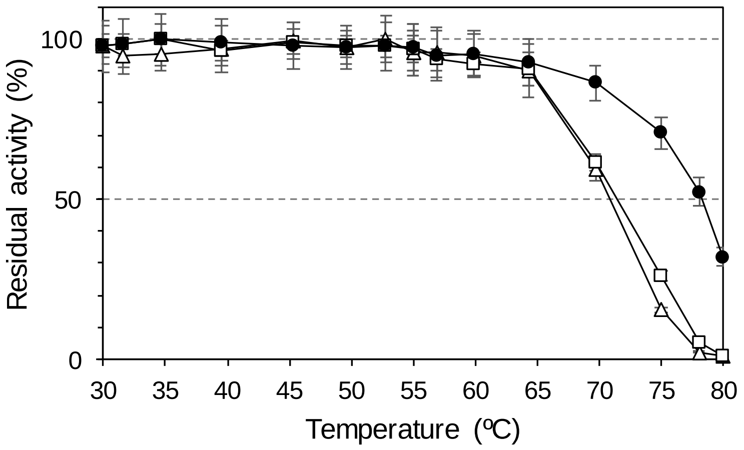

Figure S2. T_{50} (10 min) curves for domain-swap laccase (black circles), and OB1 (white squares) and 3PO (white triangles) parent laccases. Error bars indicate standard deviation for triplicates.

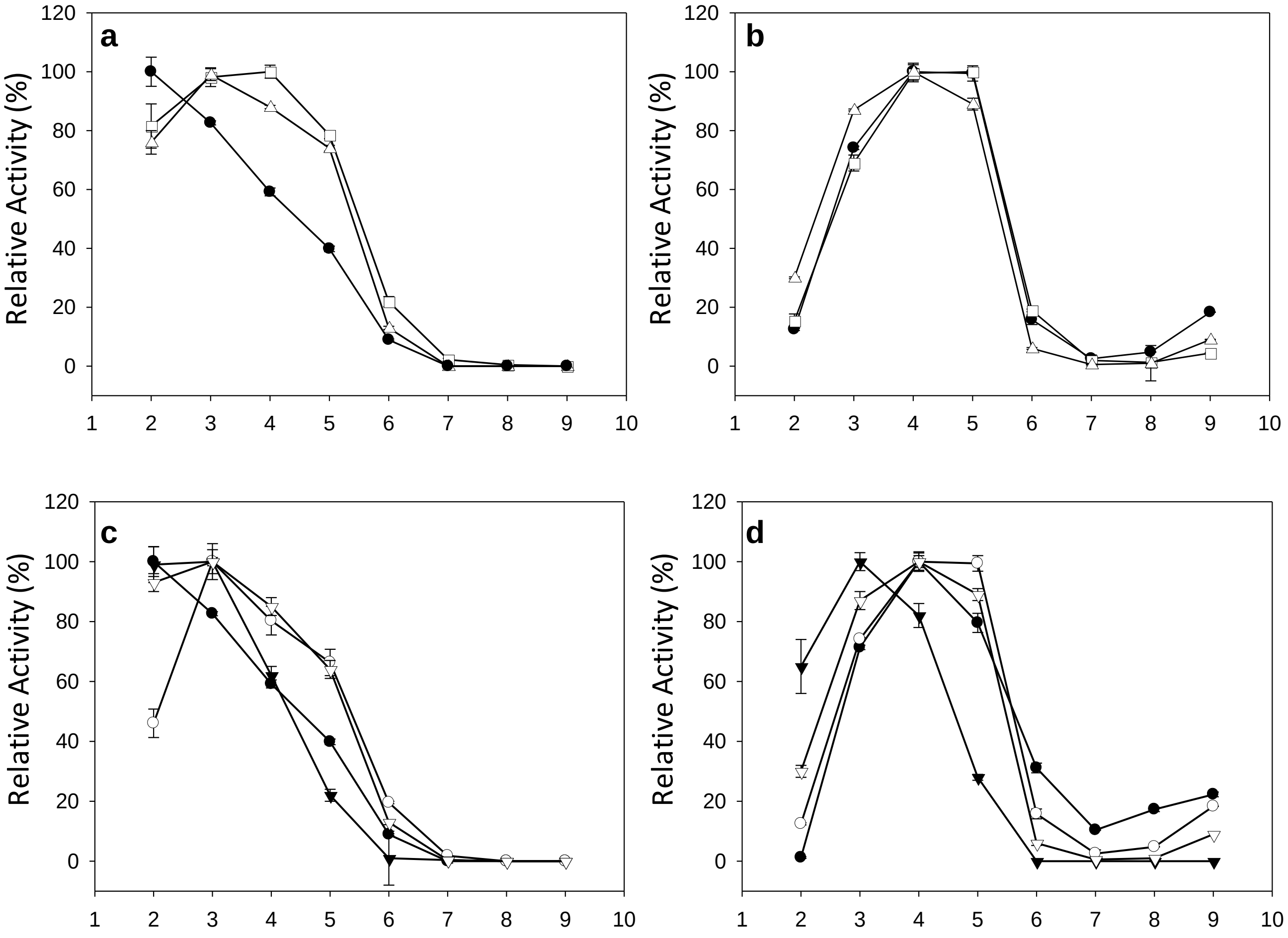

Figure S3. Optimal pH of domain-swap laccase (black circles) for ABTS (a,c) and 2,6-DMP (b,d) compared with those of parent-types OB1 (white squares) and 3PO (white triangles) (a,b), or with swap-P394H (white circles), PcL wild-type (black inverted triangles) and PcL-P394H (white inverted triangles) laccases (c,d). Mutated P394H variants were obtained to evaluate the effect of this mutation in the shifting of optimal pH to less acidic one. Error bars indicate standard deviation for triplicates.

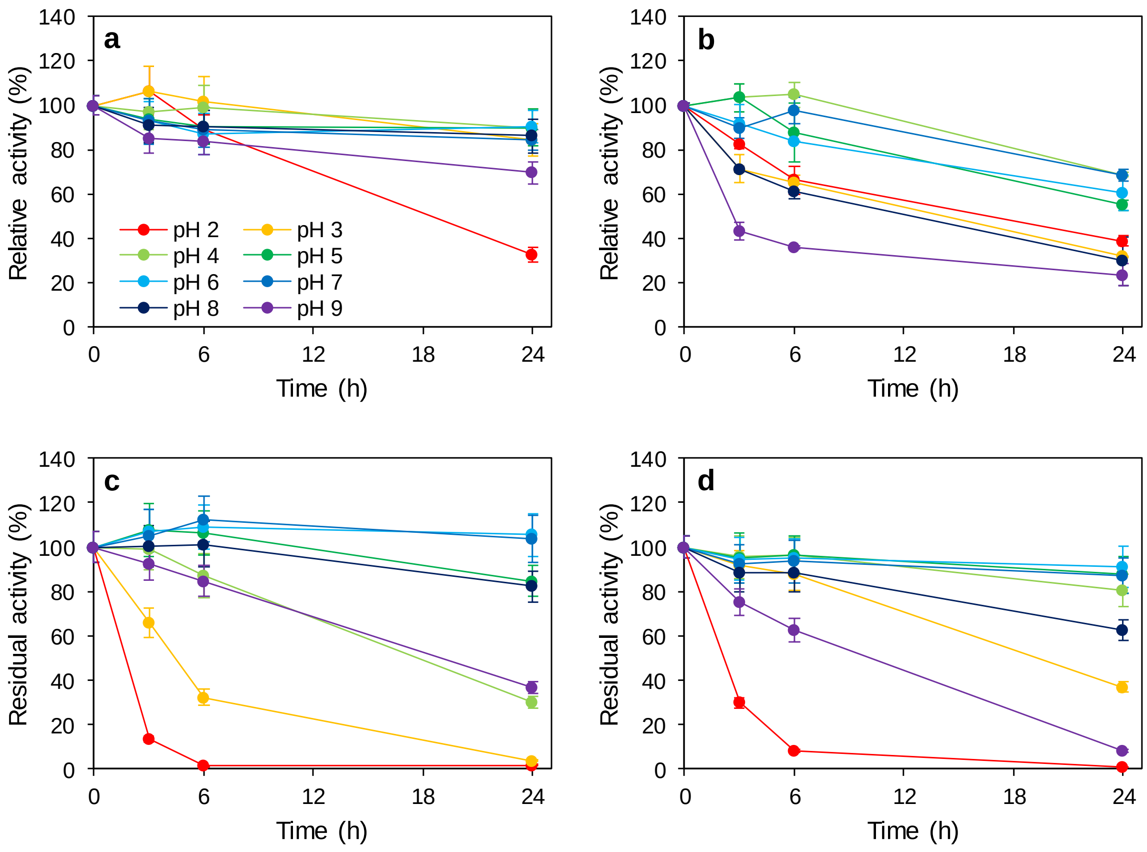

Figure S4. Residual activities for the domain-swap laccase (a), the swap-E232T/K298Q variant (b), and OB1 (c) and 3PO parent-type laccases (d) along 24 h of incubation at different pH values. Error bars indicate standard deviation for triplicates. The swap-E232T/K298Q variant was designed to remove the new putative salt-bridge inherited from D2 of 3PO laccase.

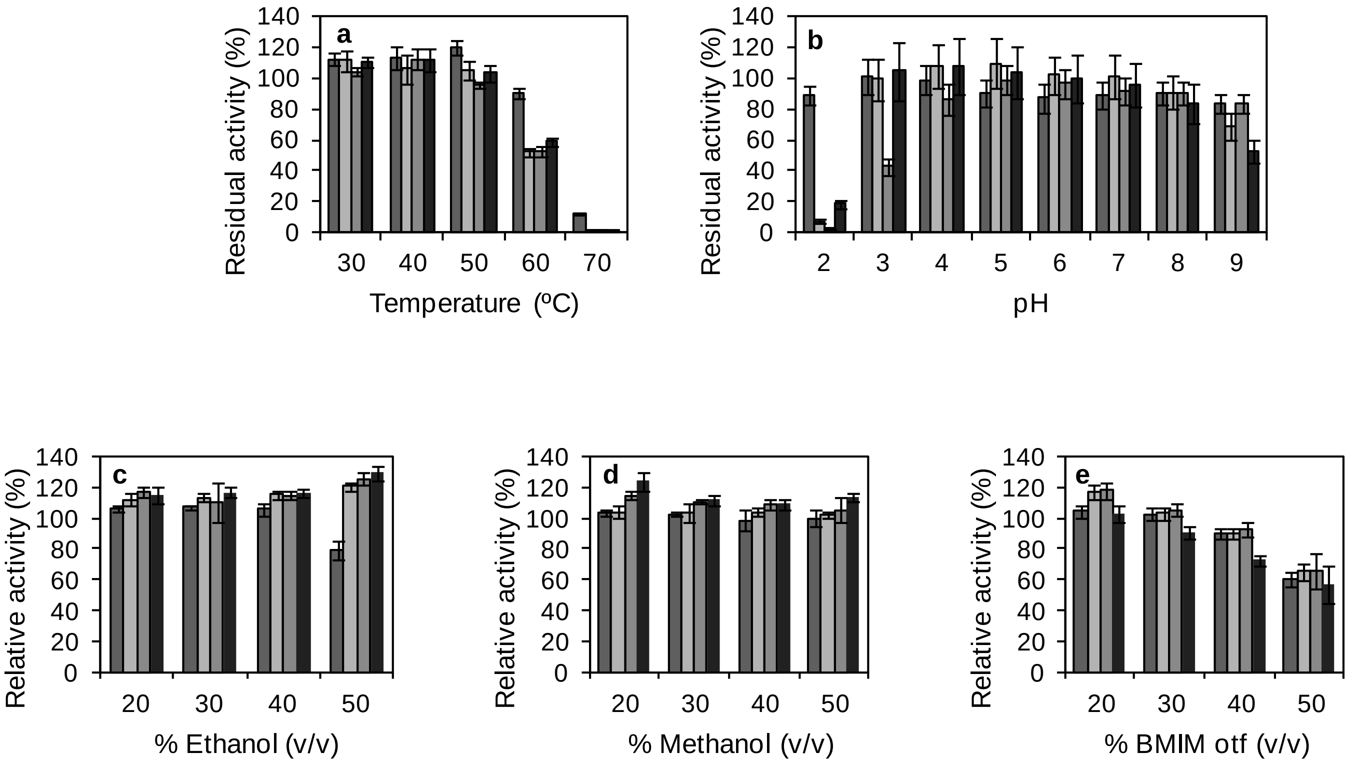

Figure S5. Long-term stabilities of domain-swap laccase compared with those of three chimeric laccases (3A4, 7A12, 7D5) obtained in a former study by random DNA-shuffling of the same parent-types that had been selected due to high stability. Residual activities after 6-h incubation at different temperatures (a), pH (b) or concentrations of ethanol (c), methanol (d) and BMIM-otf (e) From left to right: domain-swap laccase, 3A4, 7A12, 7D5. Error bars indicate standard deviation for triplicates.

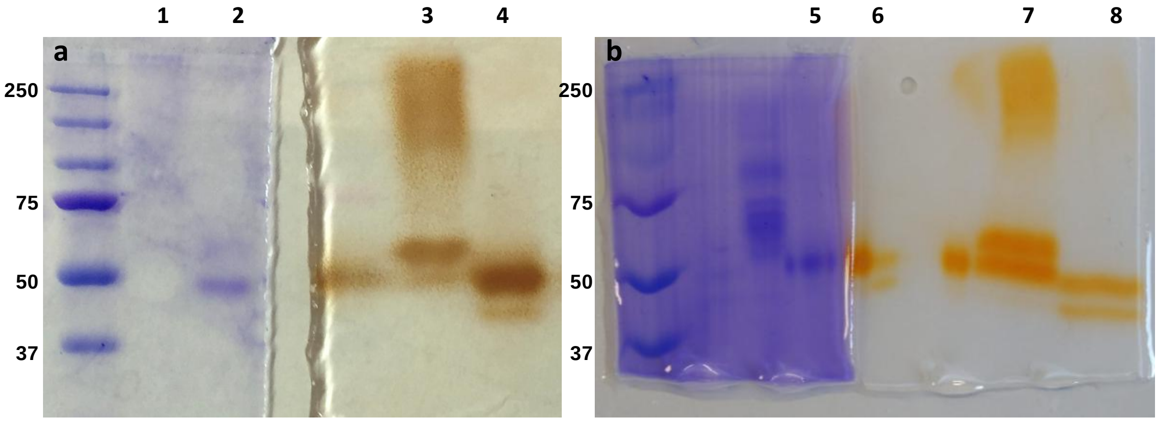

Figure S6. SDS-PAGE of native samples (non-treated with SDS, 2-mercaptoethanol or boiled) of purified domain-swap laccase (a) and of non-purified domain-swap and swap-N215G enzymes (b). Lanes 1 and 3, hyperglycosylated domain-swap isoform; Lanes 2 and 4, and 5 and 6, non-hyperglycosylated domain-swap isoform. Lane 7, non-purified domain-swap laccase; Lane 8, non-purified swap-N215G. All samples were loaded in the same 10% acrylamide/bis-acrylamide gel and run simultaneously in Tris-glycine buffer with 1% SDS. Then, both gels were split in two and the left half was dyed with Coomasie Brilliant Blue and the right half was dyed with 3 mM DMP in 100 mM acetate buffer, pH 5. The theoretical molecular weight of the domain-swap laccase according to the amino acid sequence is 53.6 kDa.

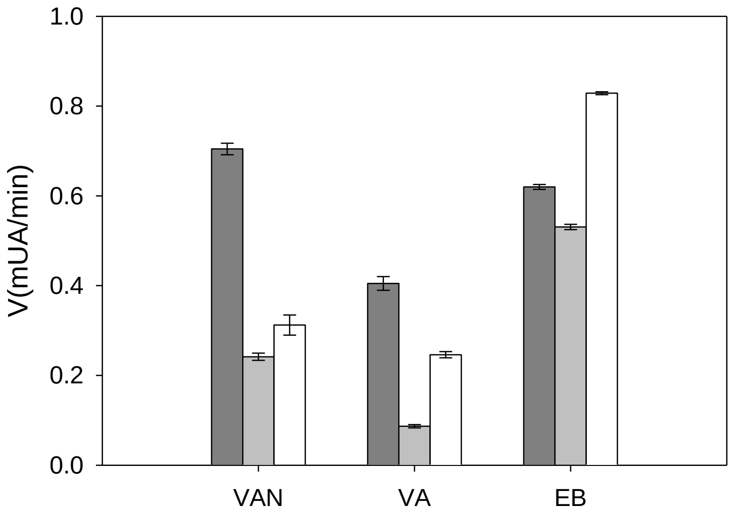

Figure S7. Oxidation rates for vanillin (VAN), violuric acid (VA), and Evans Blue (EB) by domain-swap, OB1 and 3PO laccases. Same activity units of each laccase (10 mU/well measured with 3 mM ABTS pH 3) were used in all cases for comparison. Reactions were followed in the plate reader by the change in absorbance at 410 nm for oxidation of 20 mM vanillin in 100 mM sodium acetate pH 5, 515 nm for oxidation of 20 mM violuric acid in 100 mM sodium tartrate pH 4, and 605 nm for oxidation of 50 µM Evans Blue in 100 mM sodium acetate pH 5. Error bars indicate standard deviation for triplicates.

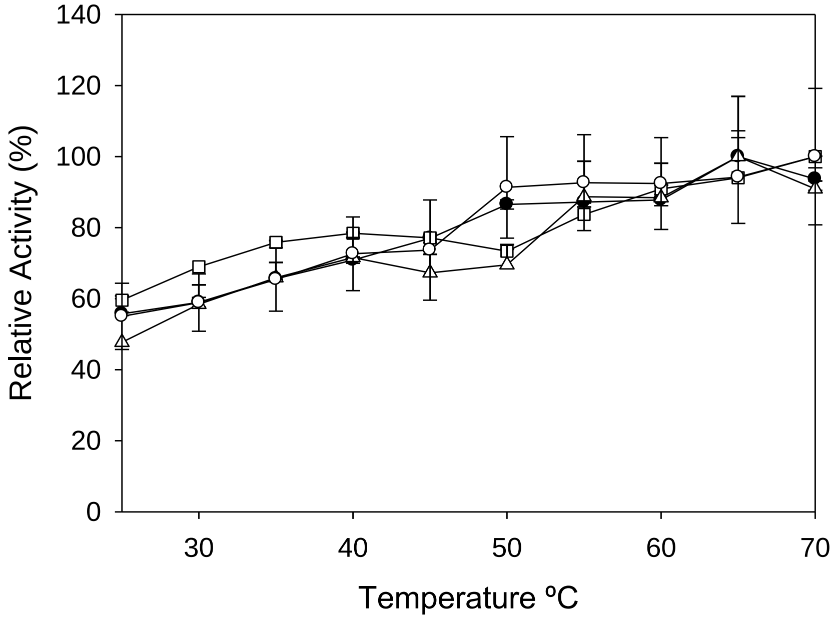

Figure S8. Activities of the purified domain-swap laccase (black circles), OB1 (white squares) and 3PO (white triangles) parent-types and hyperglycosylated isoform of domain-swap laccase (white circles) at different temperatures. Laccase activity was measured during the first minute of reaction with 3 mM ABTS in 100 mM acetate buffer pH 5 pre-incubated at the corresponding temperature. Error bars indicate standard deviation for triplicates.

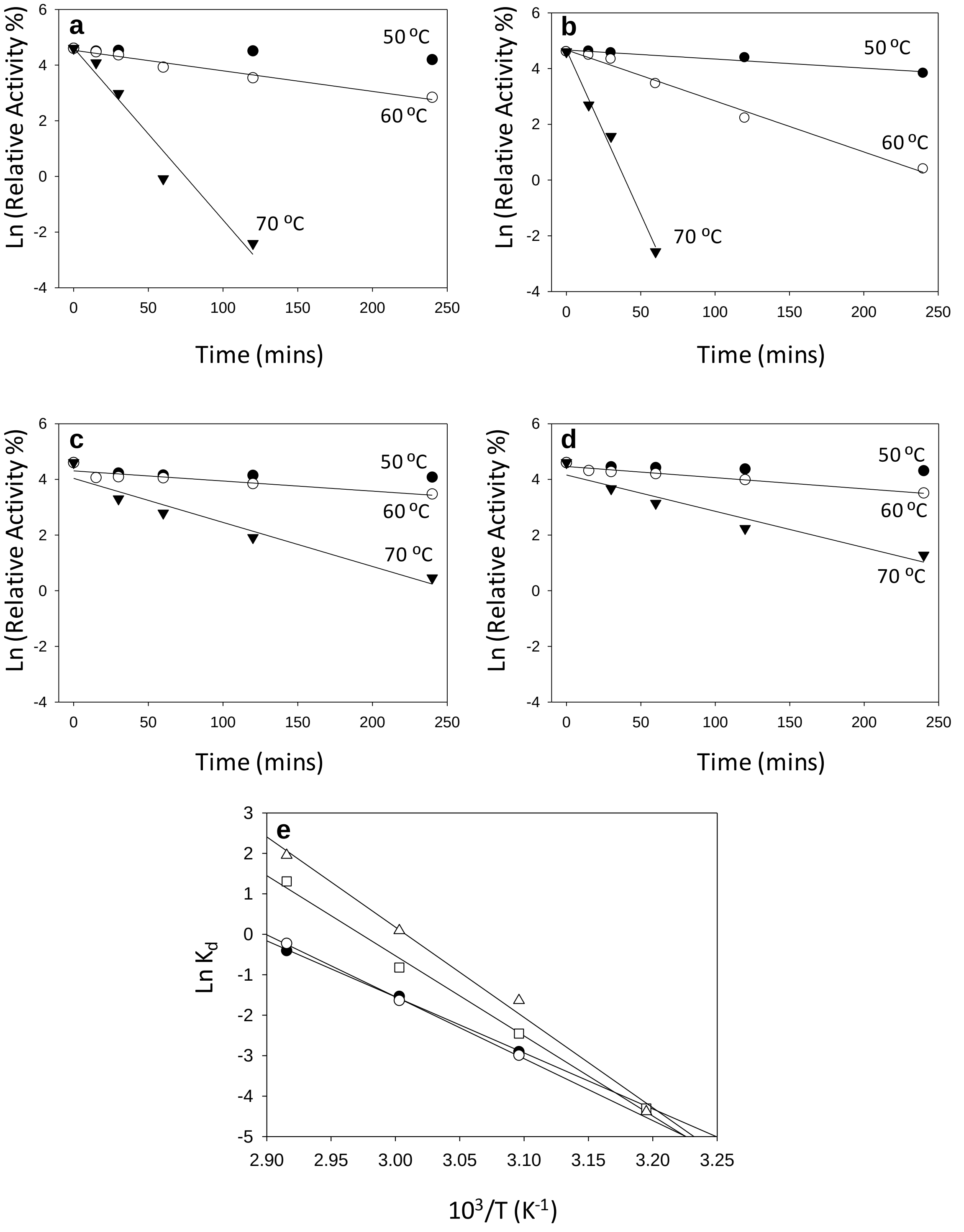

Figure S9. Thermal inactivation of OB1 (a) and 3PO (b) parent-types, domain-swap laccase (c) and hyperglycosylated domain-swap laccase (d); and Arrhenius plots (e) from the inactivation rates from (a-d) for the four laccases: domain-swap laccase (black circles), OB1 (white squares), 3PO (white triangles) and hyperglycosylated isoform domain swap laccase (white circles).

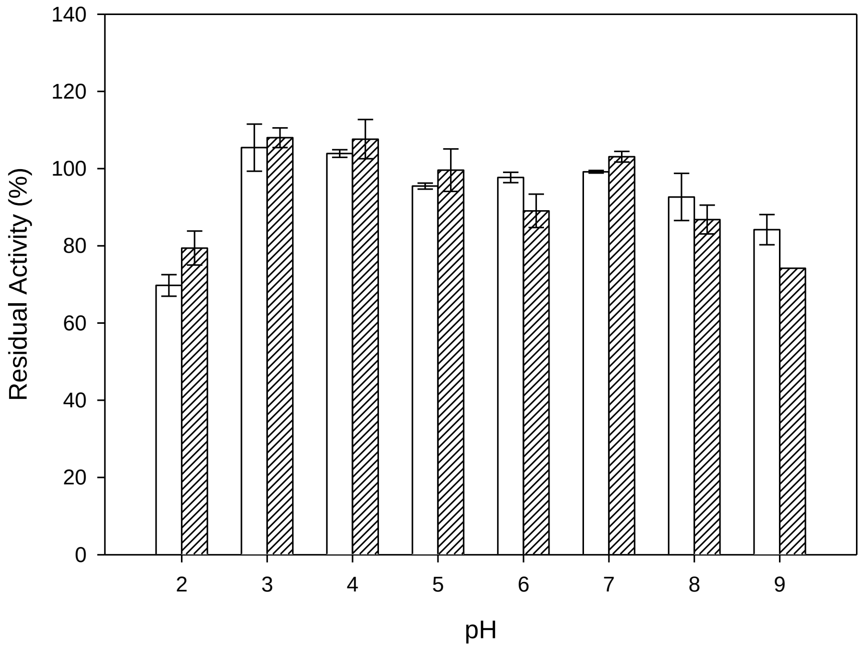

Figure S10. Residual activities of domain-swap (white bars) and swap-N215G (striped bars) laccases after 6 h incubation at different pH.
